# Hospital Wastes as Potential Sources for Multi-Drug-Resistant ESBL-Producing Bacteria at a Tertiary Hospital in Ethiopia

**DOI:** 10.3390/antibiotics13040374

**Published:** 2024-04-19

**Authors:** Mulatu Gashaw, Esayas Kebede Gudina, Wondwossen Tadesse, Guenter Froeschl, Solomon Ali, Thomas Seeholzer, Arne Kroidl, Andreas Wieser

**Affiliations:** 1 School of Medical Laboratory Sciences, Jimma University, Jimma P.O Box 378, Ethiopia; 2 CIHLMU Center for International Health, University Hospital, LMU Munich, Leopoldstrasse 5, 80802 Munich, Germany; 3 Department of Internal Medicine, Jimma University, Jimma P.O Box 378, Ethiopia; esayas.gudina@ju.edu.et; 4 Department of Medical Microbiology, Wachemo University, Hosaina P.O Box 667, Ethiopia; 5 Division of Infectious Disease and Tropical Medicine, University Hospital (LMU), 80802 Munich, Germany; 6 Saint Paul’s Hospital Millennium Medical College, Addis Ababa P.O Box 1271, Ethiopia; 7 Fraunhofer Institute for Translational Medicine and Pharmacology (ITMP), Immunology, Infection and Pandemic Research, Türkenstraße 87, 80799 Munich, Germany; 8 German Center for Infection Research (DZIF), 80802 Munich, Germany; 9 Max von Pettenkofer-Institute (Medical Microbiology), Ludwig Maximilian University of Munich, Elisabeth-Winterhalter-Weg 6, 81377 Munich, Germany

**Keywords:** hospital waste, MDR, ESBL, NDM, CTX-M, Gram-negative bacteria

## Abstract

The hospital environment is increasingly becoming an important reservoir for multi-drug-resistant (MDR) Gram-negative bacteria, posing serious challenges to efforts to combat antimicrobial resistance (AMR). This study aimed to investigate the role of hospital waste as a potential source of MDR ESBL-producing bacteria. Samples were collected from multiple sources within a hospital and its vicinity, including surface swabs, houseflies, and sewage samples. The samples were subsequently processed in a microbiology laboratory to identify potential pathogenic bacteria and confirmed using MALDI-TOF MS. Bacteria were isolated from 87% of samples, with the predominant isolates being *E. coli* (30.5%), *Klebsiella* spp. (12.4%), *Providencia* spp. (12.4%), and *Proteus* spp. (11.9%). According to the double disc synergy test (DDST) analysis, nearly half (49.2%) of the bacteria were identified as ESBL producers. However, despite exhibiting complete resistance to beta-lactam antibiotics, 11.8% of them did not test positive for ESBL production. The characterization of *E. coli* revealed that 30.6% and 5.6% of them carried *bla*CTX-M group 1 type-15 and *bla*NDM genes, respectively. This finding emphasizes the importance of proper hospital sanitation and waste management practices to mitigate the spread of AMR within the healthcare setting and safeguard the health of both patients and the wider community.

## 1. Introduction

Multi-drug-resistant (MDR) bacteria, which produce both extended spectrum beta-lactamase (ESBL) and carbapenemase, pose a significant and persistent global health threat [1]. This phenomenon has resulted in increased rates of morbidity, mortality, and escalated healthcare expenditures [1,2]. The presence of these bacteria in healthcare facilities and their surroundings further exacerbates the problem [3]. Contaminated surfaces, hospital sewage, and other environmental factors within the hospital have been identified as potential reservoirs and sources of MDR bacteria due to their close proximity to patients and healthcare workers. Furthermore, houseflies have the potential to mechanically transmit MDR bacteria to both patients and the wider community [3,4,5]. In resource-limited settings like Ethiopia, where healthcare infrastructure and waste management systems are suboptimal, the risk posed by hospital waste as a reservoir for MDR bacteria is becoming a pressing concern [1,6].

Hospital sewage serves as a conduit for the disposal of various waste materials, including fecal matter, biological wastes, biopsy specimens, clinical sample leftovers, and discarded medical supplies, potentially carrying a myriad of pathogenic bacteria [7,8]. Such sewage can contain MDR bacteria originating from infected or colonized patients, making them a reservoir for the spread of drug-resistant strains within the hospital and to the community [8]. The complex microbial niche in sewage provides opportunities for gene transfer and genetic recombination, facilitating the acquisition and spread of resistance determinants among bacteria [9,10]. Hospital environments, particularly those with inadequate sanitation and waste management practices, can attract houseflies, increasing the risk of MDR bacteria being disseminated by these insects [6,11]. They can carry bacteria on their body surfaces and within their digestive systems, facilitating their dissemination from contaminated sources to other locations [11,12].

Previous studies conducted in the same study area have reported a high prevalence of ESBL-producing bacteria and carbapenem-resistant strains among Gram-negative bacteria isolated from clinical samples [13,14,15,16,17]. The prevalence rates for ESBL producers range from 50 to 80%, while carbapenem-resistant strains range from 10 to 20% [13,14,15,16,17]. Notably, ESBL production is commonly observed in bacteria such as *E. coli*, *K. pneumoniae*, *K. variicola*, *E. cloacae*, and many others [14,15,17]. Similarly, the emergence of carbapenem resistance is frequently detected in Gram-negative bacteria such as *A. baumannii*, *P. aeruginosa*, *E. coli*, and *K. pneumoniae* [16,17]. These resistant strains have been associated with healthcare-associated infections, posing a serious threat to effective antimicrobial therapy [18]. As a result, they contribute to increased morbidity, mortality, and healthcare costs [18,19].

Jimma Medical Center, located in Ethiopia, is a tertiary hospital that serves as a referral center for the southwest region of the country and plays a crucial role in providing essential healthcare services to a substantial population [20]. However, the potential contribution of hospital waste to the spread of MDR bacteria in this setting remains poorly understood. Therefore, understanding the dynamics and sources of MDR bacterial isolates from hospital sewage, houseflies, and environmental samples provides valuable insights into the prevalence, genetic characteristics, and potential transmission routes of drug-resistant bacteria within healthcare settings and, more importantly, to the community. Thus, based on the evidence, appropriate infection control measures can be implemented to prevent their spread and reduce the burden of MDR infections. Therefore, this study aimed to provide insights on potential reservoirs for MDR and ESBL-positive pathogenic Gram-negative bacteria within the environment of Jimma Medical Center.

## 2. Results

### 2.1. Proportion of Bacterial Growth

**Figure 1:**
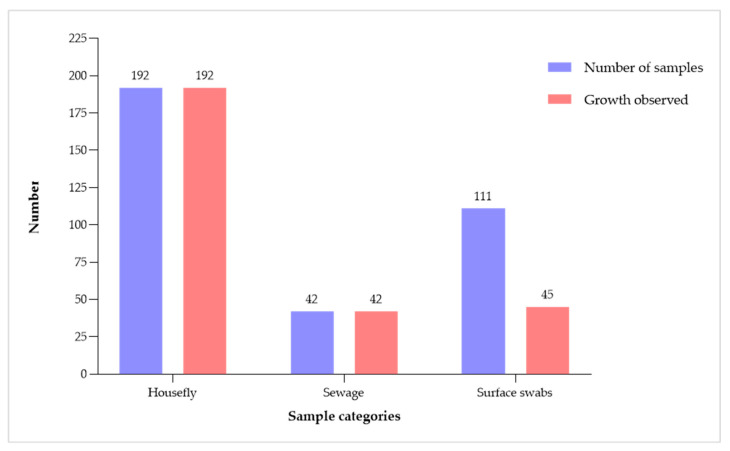
The proportion of aerobic bacterial growth obtained from housefly, sewage, and surface swab samples.

The microbiological analysis revealed the presence of potential pathogenic bacteria in samples obtained from houseflies, hospital rooms and medical device surface swabs, and sewage samples. A total of 345 samples, including 111 surface swabs and 42 sewage samples collected in 2019 and 192 housefly samples collected in 2021, were examined. The overall isolation rate was 80.9% (95% CI: 77.2% to 84.6%), with a 100% isolation rate from housefly and sewage samples. However, potentially pathogenic Gram-negative bacteria were isolated from 40.5% (*n* = 45) of hospital rooms and medical device surface swab samples (Figure 1).

### 2.2. Profile of Isolated Gram-Negative Bacteria

Further analysis of the bacterial isolates revealed a diverse range of species in surface swabs, housefly, and sewage samples. A total of 37 different species of bacteria were identified in housefly samples, while 23 species were isolated in sewage samples and 11 species in surface swabs. Among the housefly samples, *Providencia* species (20.7%) were the most frequently isolated bacteria, followed by *Proteus* species (18.6%), *E. coli* (14.9%), and *Klebsiella* species (11.2%). *E. coli* (60%), *Aeromonas* species (15.6%), and *Acinetobacter* species (8.9%) were the predominant isolates in surface swabs. In sewage samples, *E. coli* (52.1%), *Klebsiella* (19.1%), and *Acinetobacter* species (9.6%) were frequently identified. However, it is noteworthy that MDR *E. coli*, *Klebsiella*, *Acinetobacter*, and *Enterobacter* species were consistently isolated from all sample types. Despite the consistent presence of these bacterial strains across all sample types, their prevalence and abundance varied (Table 1).

### 2.3. Antibiotic Resistance Patterns

The results of the antibiotic susceptibility tests conducted on bacteria from all sample types combined revealed a significant level of resistance to several antibiotics. Specifically, a high rate of resistance was observed against cefuroxime, ampicillin, amoxicillin-clavulanic acid, piperacillin, and cefotaxime, with 100%, 61%, 44%, 42.2%, and 41.1%, respectively. Conversely, a low rate of resistance was observed against meropenem, amikacin, and piperacillin-tazobactam, representing 3.1%, 3.1%, and 8.6%, respectively. Furthermore, the double disc synergy test revealed that nearly half (49.2%) of the Gram-negative bacterial isolates were ESBL producers. A high proportion of ESBLs was observed in species such as *Acinetobacter*, *Proteus*, and *Providencia*, as indicated in Table 2. In general, an alarming level of resistance, ranging from 30% (in gentamicin) to 61% (in ampicillin), was observed to commonly used antibiotics, including beta-lactams, fluoroquinolones, and aminoglycosides, in Gram-negative bacteria isolated from various environmental samples of the medical center.

### 2.4. Molecular Epidemiology of ESBLs and Carbapenemase Expression in E. coli Strains

The findings of this study showed a high rate of ESBL- and carbapenemase-encoding genes among *E. coli* strains obtained from surface swabs, housefly, and sewage samples. A total of 66 *E. coli* strains were included in the analysis, and the presence of ESBL- and carbapenemase-encoding genes was determined using DNA microarray technology. The results revealed that 37.9% (*n* = 41) of the *E. coli* isolates exhibited at least one ESBL-encoding gene, with the predominant variant being CTX-M group 1 type-15. Additionally, 5.6% (*n* = 6) of the *E. coli* isolates carried carbapenemase genes, solely *bla*NDM. Among carbapenemase-encoding genes, five of them were found in housefly samples and the remaining one gene was detected from a surface swab. Similarly, a high rate of ESBL genes (43.9%) was detected in *E. coli* strains obtained from houseflies. However, 62.2% of the *bla*TEM genes were found in *E. coli* strains obtained from sewage samples (Table 3).

## 3. Discussion

This study revealed that bacterial isolates were present in all sewage and housefly samples, as well as in 40.5% of surface swabs. Although the proportion of bacteria detected in surface swab samples was lower compared to housefly and sewage samples, it still indicates a substantial presence of bacteria that could serve as potential sources of infections within the healthcare facility. In our study, we identified a diverse range of antibiotic-resistant bacterial isolates, including *E. coli*, *Klebsiella* spp., *Providencia* spp., *Proteus* spp., *Enterobacter* spp., *Acinetobacter* spp., *Morganella morganii*, and many others, in all categories of samples. It is worth noting that a substantial proportion of these bacteria are known to be pathogenic, or at least facultative pathogens, and have been associated with healthcare-associated infections [21,22]. This emphasizes the potential role of the environment, as well as houseflies, in perpetuating the spread of MDR pathogens, not only among patients, but to the wider community [6,23,24].

The microbiological analysis of surface swabs and sewage samples exhibited a wide array of bacterial strains, including MDR ESBL strains. Therefore, sewage was only streaked and analyzed with aerobic culture, so only the most prevalent aerobic Gram-negative bacteria would be detected. The molecular characterization of *E. coli* strains from these samples revealed the presence of acquired carbapenemase- and ESBL-encoding genes, such as (*bla*NDM) (1), CTX-M group1 type-15 (17) and CTX-M group 1, ND (2), and *bla*TEM, as well as AMPC-encoding genes, such as ACT/MIR and DHA (26). However, it is a common practice at the hospital to release sewage into the nearby stream without proper treatment. The high isolation rate of carbapenemase and ESBL bacterial strains in our study makes this practice highly hazardous. Additionally, in the hospital rooms, the floors are only mopped/cleaned twice daily with water and soap. In cases of suspected visible contamination, a 5% sodium hypochlorite solution diluted in water is used for cleaning. Such inadequate treatment and cleaning practices increase the risk of contamination for patients, healthcare providers, and caregivers in the healthcare facility, as well as water sources and the surrounding community [25,26,27,28]. In the community, transmission could occur through direct contact with contaminated surfaces and water or indirectly through animals that have direct contact with this contaminated water and environment [29]. The implications of this finding underscore the importance of implementing an effective sewage treatment system and proper cleaning practices of the hospital rooms and medical devices to mitigate the spread of MDR bacteria and minimize the risk of infections in healthcare settings.

In this study, it was found that houseflies harbor a diverse range of bacteria, including carbapenem-resistant strains and ESBL producers. Specifically, the analysis of *E. coli* strains using DNA microarray technology revealed the presence of acquired *bla*NDM genes and various ESBL-encoding genes in five and twenty *E. coli* strains, respectively. As a result, houseflies have been recognized as potential vectors for the transmission of MDR bacteria due to their attraction to waste areas such as open sewage systems, liquid and solid waste disposal sites, waste bins, and poorly cleaned toilets [30]. These insects can carry bacteria on their bodies and in their digestive systems, enabling them to spread pathogens from contaminated sources like sewage or decaying organic matter to other surfaces, including food, within a healthcare facility [30,31]. Moreover, houseflies can transport MDR bacteria from the environment into healthcare settings or vice versa [32,33]. Hence, the detection of MDR strains in the present study serves as a crucial warning, highlighting the necessity for implementing specific hygiene precautions.

The resistance spectrum of identified bacterial strains, as well as the detected resistance-encoding genes, was found to be similar to those observed in clinical samples from the same area [17]. This highlights the potential risk of transmission and the challenges in treating patients who acquire infections caused by these MDR bacteria transmitted through the hospital environment [17,34,35]. Of particular concern is the presence of the acquired *bla*NDM gene in this study, which encodes the New Delhi metallo-beta-lactamase and confers resistance to many beta-lactam antibiotics, including carbapenems, the last-resort antibiotics used to treat severe MDR bacterial infections [36]. It is worth noting that the acquired *bla*NDM gene can be horizontally transferred to other bacteria in the environment, further contributing to the dissemination of drug resistance [37,38,39]. Therefore, the high prevalence of drug-resistant bacteria in these samples underscores the urgent need for effective infection prevention and control strategies, including stringent hygiene practices and proper waste management to minimize bacterial contamination in areas prone to housefly infestation, such as toilets, sewage systems, waste bins, and the designated areas for liquid and solid waste disposal in healthcare facilities.

This study has limitations that should be considered when interpreting these findings. Firstly, it did not investigate the specific factors that contribute to the presence of MDR bacteria in hospital waste, such as the duration and storage conditions of the waste or the impact of specific infection control practices. Understanding these factors could help in identifying associated risk factors and developing appropriate waste management strategies in the hospital. Secondly, this study did not thoroughly examine the extent of the transmission risks posed by these samples, including the spread of drug-resistant bacteria to patients within the hospital and the potential dissemination to the wider community. However, we plan to perform phylogenetic analysis on these bacterial strains and compare them to patient isolates [17]. Thirdly, we conducted molecular analysis to detect resistance-encoding genes on the most prevalent species, *E. coli* only. As a result, this part of the findings may not reflect the distribution of all resistance-encoding genes in other bacterial clades obtained from surface swabs, houseflies, and sewage. Furthermore, sewage was not analyzed using filtration and enrichment techniques. Thus, the real load of MDR bacteria in sewage will be higher than described in this study once the sensitivity of isolation is improved here. We made an intentional decision for this process to limit this study to the most prevalent and most problematic isolates. In depth analysis of the sewage is beyond the scope of this manuscript and is planned for future projects.

## 4. Materials and Methods

### 4.1. Description of the Hygiene Practice in Study Area

Hospital hygiene procedures at JMC include floor mopping/cleaning conducted twice daily as part of routine tasks by janitors in the wards, waiting areas, and corridors. The cleaning of windows and tiles is performed once a week. However, these cleaning activities lacked specific protocols and typically involved the use of detergent-based products, soap, or a diluted solution of 5% sodium hypochlorite (bleach) mixed with water at a ratio of 1:10. The diluted bleach solution was mainly used in areas with frequent contamination within the facility. The solid waste of the hospital is disposed of in open or closed waste bins without undergoing proper treatment, such as autoclaving. Then, the waste is transported to an incineration facility twice daily (morning and evening). It is stored there a day prior to incineration and left open, which can lead to the attraction of houseflies (Figure S1). Furthermore, the liquid waste and sewage system of the hospital are directly released into a nearby stream without undergoing any treatment, such as chemical inactivation, filtration, or UV irradiation, prior to discharge.

### 4.2. Study Design, Area, and Period

A cross-sectional study was conducted to assess the extent and distribution of MDR ESBL pathogenic gram-negative bacteria on surfaces, sewage, and houseflies at JMC during two specific periods: May to September 2019 and June to October 2021. To avoid bias, neither the janitors nor the healthcare providers were informed about the environmental sampling, which took place at random intervals during working days. Surface swab samples were collected from various wards within JMC, including the intensive care units (ICUs) and the operating theatres, as well as the recovery rooms. Additionally, the inpatient units, such as the surgical, medical, gynecological, maternity, pediatric, and ophthalmology wards, were sampled. Furthermore, sewage and housefly samples were collected from different points within the hospital, encompassing patient care areas, wards, laboratories, and waste disposal sites. It is important to note that these environmental sample collections were conducted during periods when no known outbreaks caused by Gram-negative bacteria were reported.

### 4.3. Sample Collection

The surfaces surrounding the patients’ rooms and medical devices were sampled via swab. The following surfaces were chosen for sampling, if they were available for the individual patient: IV stands, inpatient floors, chairs, room sinks, walls, surgical tables, anesthesia tubes, forceps, chest tube sets, bedrails, bedside tables, toilet doorknobs, room doorknobs, electricity buttons, and cupboard knobs. Sterile cotton swabs pre-moistened in a sterile normal saline solution (0.9% NaCl) were used for sampling surfaces. At each site, an area of approximately 4 cm^2^ was swabbed in two directions at right angles to each other in a close zigzag pattern, rotating the swab during sampling to ensure that the entire surface of the swab was used according to the guidelines [40]. Sewage samples were collected by spot sampling methods using a wide-mouth container directly from the manholes. A total of 111 surface swabs and 42 sewage samples were collected. Using a single proportion formula, 192 housefly samples from both dry and liquid waste disposal sites of the hospital were included in this study. The sample size was calculated considering a 2.5% margin of error, a 95% confidence level, and a 3.3% prevalence of ESBL-producing *E. coli* isolated from fly samples reported in a previous study [41]. The houseflies were captured using a sweeping net and dumped in one milliliter of sterile normal saline in separate sterile glass test tubes. All samples were transported to the Core Research Laboratory of Jimma University for analysis.

### 4.4. Bacteria Isolation

In the core research laboratory, the housefly external flora was collected by dipping the housefly into a tube containing 1 mL of normal saline. Then, the housefly was briefly vortexed inside the tube to detach the bacterial flora, and all the houseflies were discarded thereafter. After this, 100 µL of the sample was inoculated on MacConkey agar. Similarly, surface swabs and 100 µL sewage samples were also inoculated on MacConkey agar. All the plates were then incubated aerobically at 37 °C for 16–18 h. After an overnight incubation, the plates were inspected and if there was growth, separate colonies were selected and sub-cultured again on MacConkey agar and incubated at the same environmental conditions to get pure cultures. For the sewage samples, to purify them easily, different individual colonies were selected from the third or fourth quadrant of the inoculated plate. These selected colonies were then subcultured under similar environmental conditions. Once the pure colony was obtained, they were saved with storage media containing skimmed milk, glucose, glycerol, tryptone soya, and distilled water at −81 °C.

### 4.5. Bacterial Identification

All stored isolates were transported to the Medical Microbiology Laboratory in Munich, Germany, and identified using matrix-assisted laser desorption ionization–time of flight mass spectrometry (MALDI-TOF MS) (Bruker, Ettlingen, Germany).

### 4.6. Antibiotics Susceptibility Test

The antibiotic susceptibility testing was performed using the Kirby–Bauer disc diffusion method for 16 antibiotics, namely ampicillin (10 µg), amoxicillin-clavulanic acid (30 µg), amikacin (30 µg), ceftazidime (30 µg), ciprofloxacin (5 µg), cefotaxime (30 µg), cefuroxime (30 µg), cefepime (30 µg), cefoxitin (30 µg), gentamicin (10 µg), meropenem (10 µg), moxifloxacin (5 µg), piperacillin (100 µg), trimethoprim-sulfamethoxazole (1.25 + 23.75 µg), tobramycin (10 µg), and piperacillin-tazobactam (10 µg) (Bio-Rad, Feldkirchen, Germany), and read using the ADAGIO 93400 automated system (Bio-Rad, Feldkirchen, Germany). The readings were interpreted as resistant, intermediate (susceptible with increased exposure), or susceptible according to the respective breakpoints for every organism in the European Committee on Antimicrobial Susceptibility Testing [42].

### 4.7. Extended Spectrum β-Lactamase Detection

The phenotypic detection of ESBL production was performed for all Gram-negative isolates by a double disc synergy test (DDST) using ceftazidime and cefotaxime with amoxicillin-clavulanic acid (10 μg) on Mueller–Hinton agar [43].

### 4.8. DNA Extraction

All *E. coli* strains that showed ESBL features from DDST and/or were resistant to cefotaxime, cefepime, cefoxitin, piperacillin-tazobactam, or meropenem in the Kirby–Bauer disc diffusion antibiotic susceptibility tests were selected for genotyping. After overnight aerobic incubation on blood agar (Oxoid, Cambridge, UK) at 37 °C, three to five pure colonies were taken with an inoculating loop and suspended in nuclease-free water and extracted using a High Pure PCR template preparation kit (Roche, Mannheim, Germany) following the manufacturer’s instructions. The quantity, purity, and concentration of extracted DNA was measured by NanoDrop ND-100 (Thermo Fisher Scientific, Wilmington, NC, USA).

### 4.9. Molecular Characterization of E. coli Strains

Check-MDR CT103XL DNA microarray kits (Wageningen, The Netherlands) were used to detect and identify encoding genes for carbapenemase (IMP, VIM, KPC, NDM-1, SPM, OXA-23 like, OXA-24 like, OXA-48 like, and OXA-58 like), AmpC-type β–lactamase (ACC, ACT, CMY, DHA, FOX, MIR, and MOX), ESBL (cefotaximase-Munich (CTX-M type)), GES, VER, PER, BEL, Temoneira β-lactamase (TEM), and sulfhydryl (SHV) variant encoding genes using the DNA microarray technique [44].

### 4.10. Data Quality Assurance

To ensure the reliability of the data, quality control (QC) measures were implemented throughout the entire laboratory process. Standard operating procedures (SOPs) were followed during the pre-analytical, analytical, and post-analytical stages to ensure the quality of the test results, thereby maintaining a high level of accuracy. Using DensiCHEK plus (BioMérieux, Craponne, France), the inoculum density of bacterial suspensions was standardized to 0.5 McFarland for all phenotypic antibiotic susceptibility tests. The Mueller–Hinton agar plates (Bio-Rad, Feldkirchen, Germany) were evenly streaked and loaded with antibiotic discs (Bio-Rad, Feldkirchen, Germany) according to the EUCAST guidelines [42]. Control strains of *Escherichia coli* ATCC 25922 and *Pseudomonas aeruginosa* ATCC 27853 were utilized to monitor the performance of antibiotic susceptibility tests.

### 4.11. Data Analysis

The data generated in the laboratory were entered into Epi-Data software version 4.6 and then analyzed using Microsoft Office 2016 Excel sheets and GraphPad Prism version 8.4.3. The findings were presented using descriptive measures, including tables, figures, and percentages.

### 4.12. Ethical Consideration

Ethical clearance was obtained from the Ethical Review Board of Jimma University, Institute of Health (protocol numbers: IHRPGO/495/2018 and IHRPGO/1087/21), and the Ethics Committee of the Medical Faculty of Ludwig-Maximilians-Universität of Munich, Germany (Opinion No: 21-0157).

## 5. Conclusions

The present study revealed a high rate of ESBL-producing Gram-negative bacteria originating from patient surroundings and the hospital environment, including houseflies caught in the hospital vicinity, as well as sewage samples. Moreover, the detection of carbapenemase- and beta-lactamase-encoding genes was observed in *E. coli* strains, with a predominant presence of *bla*NDM and *bla*CTX-M group 1, respectively. The isolation rate of MDR bacteria from the houseflies was remarkable. Therefore, the implementation of rigorous waste management and housefly control practices in and around healthcare facilities is crucial to minimize the transmission of these resistant bacteria to patients and the community at large. This includes the regular and thorough cleaning of surfaces and medical devices, along with the proper segregation, handling, and disposal/inactivation of hospital waste, particularly those with the potential for bacterial contamination. There is also a dire need for proper sewage treatment, given the total absence, especially for hospital wastewater.

## Figures and Tables

**Table 1:** The distribution of aerobic bacteria isolated from hospital rooms and medical device surface swabs, housefly, and sewage samples at a tertiary hospital in Ethiopia.

Bacteria	Housefly		Surface Swabs		Sewage		Total	
	*n*	%	*n*	%	*n*	%	*n*	%
***E. coli***	32	29.6	27	25.0	49	45.4	108	30.4
***Klebsiella* species**	24	54.5	2	4.5	18	40.9	44	12.4
***Providencia* species**	44	100	-	-	-	-	44	12.4
***Proteus* species**	40	95.2	2	4.8	-	-	42	11.8
***Enterobacter* species**	16	69.6	1	4.3	6	26.1	23	6.5
***Acinetobacter* species**	6	31.6	4	21.1	9	47.4	19	5.4
***M. morganii***	14	100	-	-	-	-	14	3.9
***Aeromonas* species**	1	NA	7	NA	2	NA	10	2.8
***Kluyvera* species**	7	NA	-	-	2	NA	9	2.5
***R. ornithinolytica***	7	NA	-	-	2	NA	9	2.5
***W. chitiniclastica***	9	NA	-	-	-	-	9	2.5
***C. freundii***	5	NA	-	-	-	-	5	1.4
***Pantoea* species**	2	NA	-	-	1	NA	3	0.8
***P. gergoviae***	1	NA	-	-	2	NA	3	0.8
***E. hermannii***	1	NA	-	-	1	NA	2	0.6
***L. adecarboxylata***	1	NA	-	-	1	NA	2	0.6
***C. sakazakii***	-	-	-	-	1	NA	1	0.3
***E. fergusonii***	-	-	1	NA	-	-	1	0.3
***Hafnia alvei***	1	NA	-	-	-	-	1	0.3
***I. indica***	1	NA	-	-	-	-	1	0.3
***M. wisconsensis***	1	NA	-	-	-	-	1	0.3
***P. carotovorum***	1	NA	-	-	-	-	1	0.3
***P. putida***	1	NA	-	-	-	-	1	0.3
***Salmonella* species**	-	-	1	NA	-	-	1	0.3
***S. maltophilia***	1	NA	-	-	-	-	1	0.3
**Total**	216	60.8	45	12.7	94	26.5	355	100

**Key**: NA: not applicable, percentage was not calculated if the total number of bacterial isolates was less than 14.

**Table 2:** The proportion of antibiotic-resistant Gram-negative bacteria obtained from surface swabs, housefly, and sewage samples at a tertiary hospital in Ethiopia.

Antibiotics	*E. coli*	*Klebsiella* spp.	*Providencia* spp.	*Proteus* spp.	*Enterobacter* spp.	*Acinetobacter* spp.	Ohers	Total
**AMP**	73.1	100	86.4	81.0	95.7	100	91.3	61.0
**PIP**	65.7	100	61.4	59.5	60.9	IE	59.6	42.2
**AMC**	38	43.2	100	21.4	91.3	100	64.3	44.0
**TZP**	19.4	31.8	9.1	0	17.4	IE	17.0	8.6
**CXM**	100	100	-	100	-	100	100	100
**CTX**	34.3	50.0	65.9	64.3	69.6	100	44.6	41.1
**CAZ**	31.5	47.7	59.1	26.2	56.5	-	40.4	27.9
**FEP**	34.3	40.9	40.9	64.3	65.2	-	42.1	30.3
**FOX**	13.9	13.6	15.9	0	100	-	46.4	18.5
**MEM**	24.1	2.3	0	0	4.3	31.6	4.3	3.1
**MXF**	35.2	38.6	61.4	76.2	56.5	-	52.2	34.7
**CIP**	30.6	40.9	47.7	66.7	30.4	100	37.5	33.9
**TM**	20.4	31.8	34.1	66.7	47.8	47.4	42.6	29.7
**GM**	16.7	31.8	36.4	66.7	65.2	52.6	32.6	30.1
**AN**	1.9	2.3	0	4.8	8.7	5.3	8.5	3.1
**SXT**	43.5	47.7	61.4	71.4	56.5	57.9	50.0	38.7
**ESBL**	38.9	43.2	61.4	64.3	52.2	68.4	37.5	49.2

**Key**: AMP, ampicillin; AMC, amoxicillin/clavulanic acid; PIP, piperacillin; TZP, piperacillin-tazobactam; CXM, cefuroxime; CTX, cefotaxime; CAZ, ceftazidime; FEP, cefepime; FOX, cefoxitin; MEM, meropenem; MXF, moxifloxacin; CIP, ciprofloxacin; GM, gentamicin; TM, tobramycin; AN, amikacin; SXT, sulfamethoxazole-trimethoprim; ESBL, extended spectrum beta-lactamase; spp., species; IE, insufficient evidence; and “-”, no breakpoints. Only resistant isolates were included in the proportion analysis, while intermediate and susceptible results were excluded from the numerator. Additionally, rare bacterial isolates that do not have breakpoints in the EUCAST guidelines were excluded from the denominator in AST analysis. The resistance patterns of specific bacterial species are found in the Supplementary Table S1.

**Table 3:** Distribution of carbapenemase- and extended-spectrum-beta-lactamase-encoding genes of *Escherichia coli* isolated from surface swab, sewage, and housefly samples at Jimma.

Types of Antimicrobial Resistance Gene	Surface Swab (*n* = 10)	Housefly (*n* = 20)	Sewage (*n* = 36)	Total	
				(*n* = 66)	%
**Carbapenemase encoding genes**	**1**	**5**	**0**	**6**	**5.6**
**NDM**	1	5	0	6	5.6
**ESBL encoding genes**	**8**	**18**	**15**	**41**	**37.9**
**CTX-M group 1 type-15**	6	15	11	33	30.6
**CTX-M group 1 type-9**	2	0	2	3	2.7
CTX-M group 1, ND *	0	1	2	3	2.8
**CTX-M group 1 type-15 + 9**	0	2	0	2	1.8
**AMPC encoding genes**	**3**	**2**	**1**	**6**	**5.6**
**CMY II (*n* = 11)**	0	1	0	1	0.9
**ACT/MIR (*n* = 10)**	3	0	0	3	2.8
**DHA (*n* = 5)**	0	1	1	2	1.9
**TEM/SHV encoding genes**	**3**	**11**	**23**	**37**	**34.3**
***bla*TEM- (WT) (*n* = 144)**	3	11	22	36	33.4
***bla*TEM-104K + 164C (*n* = 1)**	0	0	1	1	0.9

**Key**: *—no specified CTX-M group-1, subtype enzymes.

## Data Availability

The data are available from the corresponding author upon reasonable request.

## Supplementary Materials

The following supporting information can be downloaded at: https://www.mdpi.com/article/10.3390/antibiotics13040374/s1, Figure S1: Solid waste disposal practice at Jimma Medical Center, a tertiary referral hospital in Ethiopia; Table S1: The proportion of antibiotic resistant Gram-negative bacterial species obtained from surface swabs, housefly, and sewage samples at a tertiary hospital in Ethiopia.
