# Supplementary material for: Hospital Wastes as Potential Sources for Multi-Drug-Resistant ESBL-Producing Bacteria at a Tertiary Hospital in Ethiopia
Source: Antibiotics (Basel). 2024 Apr 19;13(4):374. doi: 10.3390/antibiotics13040374 (PMC11047370; doi:10.3390/antibiotics13040374)
Supplement: Supplementary file 1 [file antibiotics-13-00374-s001.zip › Supplementary Table S1.pdf]

Supplementary Table S1. The proportion of antibiotic resistant Gram-negative bacterial species obtained from surface swabs, housefly, and sewage samples at a tertiary hospital in Ethiopia.

**Microorganism**	**AMP**	**AMC**	**AN**	**CAZ**	**CIP**	**CTX**	**CXM**	**FEP**	**FOX**	**GM**	**MEM**	**MXF**	**PIP**	**SXT**	**TM**	**TZP**
A. baumannii (n = 7)	7	7	1	-	7	7	7	-	-	5	4	-	IE	5	5	IE
A. calcoaceticus (n = 1)	0	0	0	-	1	1	1	-	-	0	0	-	IE	0	0	IE
A. johnsonii (n = 2)	-	-	0	-	2	-	2	-	-	1	0	-	IE	2	1	IE
A. nosocomialis (n = 1)	0	0	0	-	1	1	1	-	-	1	1	-	IE	1	0	IE
A. pittii (n = 2)	2	2	0	-	2	2	2	-	-	0	1	-	IE	0	0	IE
A. baylyi (n = 3)	3	-	0	-	3	-	3	-	-	1	0	-	IE	1	1	IE
A. towneri (n = 1)	-	-	0		1	-	1	-	-	0	0	-	IE	0	0	IE
A. ursingii (n = 2)	-	-	0	-	2	-	2	-	-	2	0	-	IE	2	2	IE
E. asburiae (n = 3)	3	3	0	0	2	2	-	0	3	2	0	2	0	0	0	0
E. cloacae (n = 13)	13	13	2	10	4	12	-	13	13	11	1	8	12	11	10	4
E. kobei (n = 1)	1	1	0	1	1	1	-	1	1	0	0	1	1	1	0	0
E. bugandensis (n = 4)	4	4	0	2	1	1	-	1	4	2	0	2	1	1	1	0
E. xiangfangensis (n = 2)	1	0	0	0	0	0	-	0	2	0	0	0	0	0	0	0
E. coli (n = 108)	73.1	65.7	30.0	19.4	100	34.3	31.5	34.3	13.9	24.1	35.2	30.6	20.4	16.7	1.9	43.5
K. oxytoca (n = 10)	10	2	0	4	2	4	10	1	1	2	0	4	10	5	2	1
K. pneumoniae (n = 21)	100	80.9	4.8	76.2	61.9	80.9	100	76.2	23.8	57.1	4.8	61.9	100	66.7	57.1	57.1
K. variicola (n = 13)	13	0	0	1	3	1	13	1	0	0	0	0	13	2	0	0
P. alcalifaciens (n = 5)	3	5	0	3	4	3	-	3	2	1	0	4	3	3	1	0

P. rettgeri (n = 21)	90.5	100	0	47.6	47.6	61.9	-	42.9	14.3	47.6	0	61.9	57.1	52.4	28.6	4.8
P. stuartii (n = 17)	16	17	0	13	7	13	-	6	2	5	0	10	12	13	8	3
P. vermicola (n = 1)	0	1	0	0	0	0	-	0	0	0	0	0	0	0	0	0
P. hauseri (n = 4)	4	0	0	0	1	0	4	0	0	0	0	2	0	0	1	0
P. mirabilis (n = 31)	93.5	29.0	6.5	35.5	80.6	80.6	100	80.6	0	83.9	0	87.1	74.2	90.3	83.9	0
P. vulgaris (n = 7)	1	0	0	0	2	2	7	2	0	2	0	3	2	2	1	0
Cronobacter sakazakii (n = 1)	1	0	0	1	0	1	-	1	0	1	0	0	1	1	1	0
Raoultella ornithinolytica (n = 9)	9	3	2	6	7	6	9	7	0	3	0	7	9	7	7	1
Aeromonas caviae (n = 4)	-	4	-	1	0	-	-	1	4	-	-	-	-	1	-	-
Aeromonas hydrophila (n = 5)	-	5	-	0	0	-	-	0	5	-	-	-	-	0	-	-
Aeromonas veronii (n = 1)	-	1	-	0	0	-	-	0	1	-	-	-	-	0	-	-
Citrobacter freundii (n = 5)	5	5	0	4	3	4	-	3	5	2	0	3	4	4	2	4
Escherichia fergusonii (n =1)	1	0	0	0	0	0	-	0	0	0	0	0	0	0	0	0
Escherichia hermannii (n = 2)	2	0	0	0	2	2	-	2	0	2	0	2	2	2	2	0
Kluyvera ascorbata (n = 4)	3	0	0	1	0	2	-	2	0	1	0	1	2	3	1	0
Kluyvera crypcrescens (n = 5)	4	2	0	2	1	3	-	3	0	2	0	2	3	3	2	1
Leclercia adecarboxylata (n = 2)	1	0	0	1	0	1	-	1	0	0	0	0	1	1	0	0
Moellerella wisconsensis (n = 1)	1	1	0	1	0	1	-	0	1	0	0	0	1	1	0	0
Morganella morganii (n = 14)	14	14	0	4	5	4	-	2	9	2	0	8	3	4	2	0
Pseudomonas putida (n = 1)	-	-	0	1	1	-	1	1	-	IE	1	-	1	-	1	1
Salmonella species (n = 1)	0	0	1	0	-	0	-	0	0	1	0	0	0	0	1	0

Stenotrophomonas maltophilia (n = 1)	1	1	1	1	1	1	1	1	1	1	1	1	1	1	1	1

Key: AMP, ampicillin; AMC, amoxicillin/clavulanic acid; PIP, piperacillin; TZP, piperacillin-tazobactam; CXM, cefuroxime; CTX, cefotaxime; CAZ, ceftazidime; FEP, cefepime; FOX, cefoxitin; MEM, meropenem; MXF, moxifloxacin; CIP, ciprofloxacin; GM, gentamicin; TM, tobramycin; AN, amikacin; and SXT, sulfamethoxazole- trimethoprim; Percentage is not calculated if the denominators are less than 20.
