# Supplementary figures and images for: Hospital Wastes as Potential Sources for Multi-Drug-Resistant ESBL-Producing Bacteria at a Tertiary Hospital in Ethiopia
Source: Antibiotics (Basel). 2024 Apr 19;13(4):374. doi: 10.3390/antibiotics13040374 (PMC11047370; doi:10.3390/antibiotics13040374)

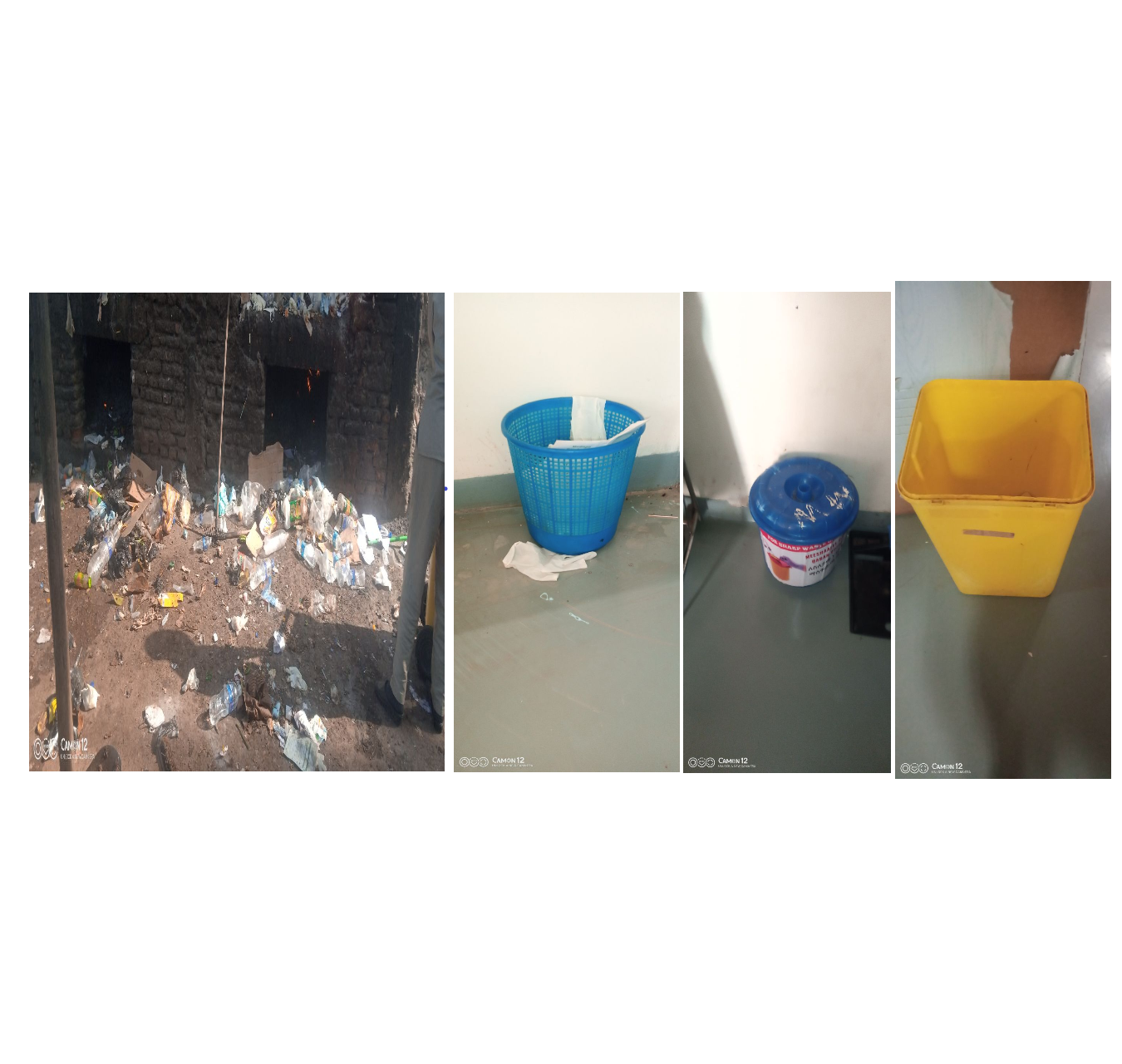

Supplement: Supplementary file 1 [file antibiotics-13-00374-s001.zip › Figure S1.png]
